# Racism, early psychosis, and institutional contact: A qualitative study of Indigenous experiences

**DOI:** 10.1177/00207640231195297

**Published:** 2023-09-04

**Authors:** Jenni Manuel, Suzanne Pitama, Mauterangimarie Clark, Marie Crowe, Sue Crengle, Ruth Cunningham, Sheree Gibb, Frederieke S Petrović-van der Deen, Richard J Porter, Cameron Lacey

**Affiliations:** 1 Māori/Indigenous Health Innovation, University of Otago Christchurch, New Zealand; 2 Department of Psychological Medicine, University of Otago Christchurch, New Zealand; 3 Department of Preventive and Social Medicine, University of Otago, Dunedin School of Medicine, New Zealand; 4 Department of Public Health, University of Otago Wellington, Newtown, Wellington, New Zealand; 5 Te Whatu Ora Waitaha, New Zealand

**Keywords:** Indigenous, ethnicity, psychosis, racism, institutions, risk

## Abstract

**Background:**

There is evidence of Indigenous and ethnic minority inequities in the incidence and outcomes of early psychosis. Racism has been implicated as having an important role.

**Aim:**

To use Indigenous experiences to develop a more detailed understanding of how racism operates to impact early psychosis outcomes.

**Methods:**

Critical Race Theory informed the methodology used. Twenty-three Indigenous participants participated in four family focus group interviews and thirteen individual interviews, comprising of 9 Māori youth with early psychosis, 10 family members and 4 Māori mental health professionals. An analysis of the data was undertaken using deductive structural coding to identify descriptions of racism, followed by inductive descriptive and pattern coding.

**Results:**

Participant experiences revealed how racism operates as a socio-cultural phenomenon that interacts with institutional policy and culture across systems pertaining to social responsiveness, risk discourse, and mental health service structures. This is described across three major themes: 1) selective responses based on racial stereotypes, 2) race related risk assessment bias and 3) institutional racism in the mental health workforce. The impacts of racism were reported as inaction in the face of social need, increased use of coercive practices and an under resourced Indigenous mental health workforce.

**Conclusion:**

The study illustrated the inter-related nature of interpersonal, institutional and structural racism with examples of interpersonal racism in the form of negative stereotypes interacting with organizational, socio-cultural and political priorities. These findings indicate that organizational cultures may differentially impact Indigenous and minority people and that social responsiveness, risk discourse and the distribution of workforce expenditure are important targets for anti-racism efforts.

## Introduction

A pervasive pattern of ethnic disparities in early psychosis have been established internationally (Halvorsrud et al., 2019; Seltenet al., 2020). Disparities in first episode psychosis are also well recognized in New Zealand, with non- Māori experiencing lower rates of psychotic disorders (Petrović-van der Deen et al., 2020), a higher level of social privilege for those diagnosed with psychosis (Manuel et al., 2023), and subsequent better outcomes (Cunningham et al., 2023) than Māori the Indigenous people. Differential cumulative exposure to social and environmental psychosis risk factors across the lifespan are known to contribute to these inequities, with ethnic minority and Indigenous populations being the most impacted (Jongsma et al., 2021; Morgan et al., 2019; Petrović-van der Deen et al., 2020). For Indigenous populations, colonization has shaped the social gradient of disparities, through historical and contemporary acts of dehumanization, political re-organization and misappropriation of land and other resource and subsequent intergenerational loss of wealth, culture, language and sovereignty.

Racism is now well recognized as an important social determinant of mental health (Paradies et al., 2015). It manifests in various forms and tends to be defined at different levels. This includes: 1) systemic or institutional, which is the norms embedded in culture, society and organizational practices and policy, that privilege majority populations over others, 2) interpersonal, personally mediated racism that occurs at the individual level between people and 3) internalized, racism involving intrapersonal beliefs of inferiority related to race in oppressed groups (Jones, 2000). In psychosis, racism has been identified as an important social risk factor (Lazaridou et al., 2022; Nazroo et al., 2020). This includes at the interpersonal and internalized level with clear evidence of an association between experiences of racism and discrimination enhancing psychosis risk (Anglin et al., 2014; Karlsen et al., 2005) and relevant to this, a relationship between ethnic density and discrimination (Baker et al., 2021). More broadly, there is also the understanding that systemic and institutional racism shapes and maintains the inequitable distribution of socio-environmental risk that contributes to psychosis disparities (Anglin et al., 2021).

Despite many authors theorizing how racism impacts psychosis disparities (Anglin et al., 2020; Nazroo et al., 2020), there has been limited qualitative exploration of the association between racism and psychosis. A perceived relationship between racism and psychological risk factors has been illustrated in recent qualitative research, for example, the impact of racism on identity and sense of self and experiences of racism related to persecution and subsequent paranoia (Lazaridou et al., 2022). There is also more general qualitative literature that identifies racism impacts the level of trust people have in mental health service delivery (Alang, 2019). However, in order to dismantle racism associated with psychosis, more detail regarding how it operates is required. Qualitative methods are well placed to investigate this type of nuanced detail. This study sought to answer the following research question:

What do Indigenous youth with psychosis, their families, and Indigenous mental health professionals’ working in early psychosis experiences tell us about how racism is operating to impact early psychosis outcomes?

## Methods

### Critical race theory and the researchers positioning

Critical race theory (CRT) was used as a methodological approach that informed the way the research was conducted. This framework is interested in the experiential knowledge of marginalized groups and how their experiences offer a means to examine the functions of racism in its various forms (Graham et al., 2011). Some of the key tenants of CRT is that racism is normal and that race is socially constructed (Ladson-Billings, 2021). This denotes the understanding that race in itself is not a risk factor for poor health but an indicator of exposure to risk, which is shaped through structural and institutional racism. There is also a requirement in CRT to challenge white assumptions and avoid deficit framing the problem as inherent to the marginalized group (Doharty et al., 2021). It was these frames of reference that shaped the way the research was conducted.

CRT also challenges the notion that research is objective (Doharty et al., 2021) and with this, it is acknowledged that the researchers’ positioning has impacted the qualitative research process and its outcomes. The researchers in this study have a variety of backgrounds and disciplines. These include public health, psychiatry, mental health nursing, psychology and general practice. The research group includes a mix of both Indigenous (SP, MC, SC, CL) and non-Indigenous researchers (JM, MC, RC, RJP, FSP, SG), who are partners in the Treaty of Waitangi, New Zealand’s founding document. Therefore, both parties have responsibilities to honour the Treaty’s principles. As discussed by Cram et al. (2006), in a research context this terminology can be used to describe research relationships between Māori and non-Māori researchers, with both parties having clear responsibilities and a reason to invest in the research process. It is also based on the premise that the non-Indigenous researchers are not doing research on Māori but for the benefit of Māori. There was a high level of oversight and leadership from Indigenous researchers in the research group and a clear recognition and integration of Māori values and protocols incorporated in to the study design. All the authors have a strong desire for social justice and anti-racism and this operates as a principle driver of research endeavours.

### Setting and recruitment

The research was conducted in two urban areas and one rural area in New Zealand. Recruitment occurred via clinicians in community public mental health services, including specialized early intervention in psychosis services and general adult services. Māori youth participants were required to self-identify as Māori, have had a first episode of psychosis in the previous 5 years, and be aged between 16 and 30 years old. Family members who had supported the youth through the experience of FEP were also invited to participate in a focus group with the youth; however, youth and family members had the option to be interviewed individually. Māori health professionals who worked with Māori youth with early psychosis were also invited to participate. Māori health professionals were identified by managerial staff working in the localities and approached directly by the researchers. All participants received a gift voucher in recognition of the time given to the study and interviews.

### Data collection

Each family focus group and individual interview was conducted by two researchers (JM, MC, CL), with at least one Indigenous researcher. A semi-structured interview format was used, which is consistent with both qualitative and CRT research methods given the flexibility of the approach. It allowed for the incorporation of Māori protocols, including an interview structure designed for Māori (Lacey et al., 2011). The main body of the interviews were organized around a set of predetermined topics, informed by results of a NZ national cohort study (Manuel et al., 2023). These topics were pathways and experience of treatment for early psychosis, early life experiences with a specific focus on institutional contact and responses, and experiences of contact with the range governmental systems previously identified as having a high rate of contact with Māori with FEP, such as health, criminal-justice and social services. They were broadly asked about what was helpful and was difficult about the contact they had with these services. Māori health professionals were asked to recount experiences of working with Māori youth with FEP and their families, including their interaction with other government systems associated with this work. Interviews ranged in length from 40 to 60 minutes. Interviews were audio recorded and later transcribed verbatim. All participants were given the option of reviewing their written transcripts; however, none of the participants requested to review their transcripts.

### Analysis

NVivo software was used to manage the qualitative data. Based on the need to frame the results consistent with a CRT approach, a theory driven deductive orientation was undertaken by JM in the initial stages of the analysis process. This involved the use of structural coding, described by Saldaña (2016) and more specifically by Haitana et al. (2020) in a Māori context, as the first round of coding. This method involves selecting a framework to inform the first round of coding as a means of ensuring the colonial and socio-political context of disparities are not overlooked. The World Health Organization’s (World Health Organization, 2014) framework for addressing mental health inequities was selected as it aligned with current thinking in the broader scholarly context for psychosis-based inequities. The first round of coding was based on the four main components of the framework – life course stages, systems, the macro-level context and wider society. The framework’s wider society structural code was adapted to focus on understanding how social and cultural factors influence social priorities including how services and interventions are structured and delivered. The results presented in this paper are an analysis of the wider-society data that identified participant experiences and reports of racism. The first round of coding involved deductive analysis using structural coding to identify experiences reflective of racism. This data was then inductively coded using descriptive coding methods (Saldaña, 2016). The wording of the initial codes was refined throughout the analytic process, and similar codes were clustered. Once the data was descriptively coded, a second cycle of coding used a further inductive analysis method, pattern coding to create initial sub-categories and then categories. The raw data was reviewed in relation to these sub-categories and categories to ensure a good fit.

### Ethical statement

All participants provided written informed consent. The study received approval by the University of Otago Ethics Committee (HD/18/065).

## Results

A total of 23 participants participated in four family focus group interviews (one Indigenous youth with FEP and one to four family members in each), and 13 individual interviews. This was comprised of 9 Indigenous youth with FEP [IY], 10 family members [F] and 4 Māori health professionals [HP]. The Indigenous youth were aged between 18 and 28 years, with an average age of 23 years. Six were male and three were female. Diagnoses included Bipolar Affective Disorder (*n* = 3), Psychosis Not Otherwise Specified (*n* = 3), Schizoaffective Disorder (*n* = 2) and Schizophrenia (*n* = 1).

Participant voices revealed racism was pervasive and impacted contact with multiple institutions. The results are presented across three themes, 1) selective responses based on racial stereotypes, 2) race related assessment bias and 3) institutional racism in the mental health workforce.

1. Selective responses based on racial stereotypes.

This theme reports participants’ experiences of racism across multiple systems and how it operated as a barrier to meaningful assistance or intervention that could have preventative effects. Experiences of racism were pervasive across various institutions, including social services, police, justice and health services and included examples dating back to participants’ early years. The racism described often took the form of negative stereotypes, such as the Indigenous youths being viewed as criminal, bad or ‘behavioural’. These stereotypes were then the basis for adverse or inadequate responses to their needs.

The following example demonstrates the dehumanizing nature of racism. It also illustrates how the Indigenous youth was racially stereotyped as being criminal rather than being seen as a youth in need of social assistance.

The police came and found her and there had been a burglary down the road at the same time and they said it was you, you robbed that house. She was like . . . I rang you, like I’m covered in bruises, I’ve got a black eye, I’ve been seriously beaten up, I called for help. (HP10)

Participants often described early signs of the presence of poor mental health, which was related to the social conditions that the youths had been exposed to. This was interpreted into the negative stereotypes, such as the categorization of Māori youths as being bad. Judgements made were often void of understanding the social or mental health context of their behaviour. In the following example, the youth was challenged in a way that insinuated he was aggressive. This response left the youth feeling misunderstood and reduced his motivation for the intervention on offer.

I did this anger management course and the first thing the anger management dude said – you look like you are 10-foot-tall and bullet proof and I’m like stuff you. Like, I just can’t handle it, stuff you. (IY02)

Prior to diagnosis, Māori youth, particularly males, were often perceived to be aggressive or as having behavioural issues. This deficit framing and blaming was then used as justification for inaction. This included the reasoning that behavioural issues were not within the scope of institutional responsibilities.

I think Youth Aid or some youth worker with the police as well was just kind of yep following in the footsteps of Dad. (HP24)

That’s a young Māori boy. It’s an 11-year-old Māori boy who’s not settling into school. Like, he’s getting himself into trouble. And they are like, oh, it’s behavioral. It’s not us. And then they are gone. (HP12)

Previous experiences of racism also translated to an intuitive sense of racism in future interactions with systems. The following example described how a mother could detect racism was operating when her son had difficulty getting a sickness benefit despite a medical certificate.

I think because he’s just a young boy. No-one’s there with him. You know, you are just a little – and to be a little bit fair, I was probably thinking well because your Māori you know. They just think you are just another dole bludger or something like that. (F05)

This theme demonstrates that Māori youth presenting with first episode psychosis potentially experience racism in multiple systems both prior and during treatment. The racism was in the form of negative stereotypes, which were then used as justification for institutional abdication of responsibility for responding to Māori youth presenting with early signs of poor mental health in the context of social and environmental stress.

2. Race related risk assessment bias

This theme reports participant narratives that describe the interplay between racism and mental health risk discourse and how this impacts treatments. Risk in the mental health context is conceptualized as the potential for harm (aggression, violence, drug use, self-harm and suicide) and is often viewed as something that the patient creates in an environment. There is a requirement for mental health service providers to assess the level of risk potentially posed by a person and consider how to mitigate that risk. Within this theme, participants described how negative stereotypes were carried forward into the mental health system, resulting in bias in risk assessment and subsequent differential management. Examples included a perceived increased risk of drug use and aggression rather than an emphasis on risk to self.

When I was on drugs because they would have viewed that as like a stereotypical thing that Māori does and obviously view that as the reason, they just tell you don’t take drugs and I guess that’s kind of their example to what most Māori’s do, they think that’s the problem but obviously it goes deeper than that. (IY09)

The following excerpt describes the perception of increased risk status for Māori and how this was perceived to impact decision making and treatment, including an increased use of compulsion.

I’ve noticed just in our service as well if it’s a Māori male they say more likely to be under the Mental Health Act for longer periods of time, I think because people are viewing risk as higher, I have had specific cases where it was same stuff going on and it was a Pakeha [white] female and they were treated really differently. (HP10)

The following example also describes how being Māori can be used as an indicator of risk of violence, which then preempted the planned use of force in the form of increased provision of staffing numbers.

The other one is violence. You know, Māori male violent, and so a whole lot of things go up then as well. About where you are going to see them and about who is going. Staff are going oh you need to get help for that . . . Not all the time. Well, actually, yes, if it was that scenario. Young Māori male with a history of police, they are already forming some opinion and plan to deal with it. (HP12)

Within this theme, racism was reported to impact the perceived level of risk for drug use and aggression once receiving treatment for psychosis and subsequent higher use of compulsion and force. This illustrates one of the ways racism interacts with institutional policy and practice and results in differential treatment.

3. Institutional racism in the mental health workforce

In this final theme, participants describe institutional racism operating through Māori health worker roles being undermined by a lack of adequate resourcing and support. Most participants reported that the only Māori specific interventions they received was contact with a Māori health worker. However, it was often expressed that they did not understand the Māori health workers role, which was largely related to a lack of contact time they had with them.

Yeah, she was really nice but I didn’t see her often so I didn’t really know what her role was. (IY20)

Health professional participants indicated that this was because Māori health workers had unrealistically high caseload numbers that did not enable them to consistently engage meaningfully with Māori. This undermined Māori health workers abilities to work in a way that is congruent with Māori principles that value families, relationships, and connection.

I guess the big one here is Pukenga Atawhai [Māori health worker] hours, so we only have one here like two and a tiny bit days a week, and a large part of our case load is Māori clients. So just for them to be able to have that regular interaction with her just isn’t always possible because she has so many people to see, she ends up just kind of seeing people in crisis. (HP10)

Participants also indicated there remained a lack of collegial support for Māori specific roles. This was portrayed as a lack understanding of the value and scope of their roles.

What do you mean you are a Māori Health Nurse? What’s that? You know, we are all nurses. So, there was lots of attitude stuff back then. The sad thing is, it’s actually still there. It’s just that’s a little bit hidden. But it’s actually still there. They still don’t actually get it. They don’t value it, having Māori involved. (HP12)

Within this theme participants described current Māori specific roles within the mental health system. Their descriptions indicated that these roles were under resourced, resulting in significant limitations in their ability to engage meaningfully with Māori youth.

## Discussion

In this study, participants provided examples and experiences of racism across systems, illustrating some of the ways racism operates to maintain inequitable outcomes for Indigenous youth with early psychosis. While participants described racism at the interpersonal level, their experiences illustrated how racism is a socio-cultural phenomenon that interacts with institutional policy and culture pertaining to social accountability, risk discourse and institutional service structures. At the interpersonal level, early signs of social and mental health need were interpreted as negative stereotypes including Māori youths being labelled as criminal, ‘behavioural’ or high risk. These negative stereotypes aligned with organizational culture and were used to justify inaction because they could be viewed as being out of the scope of institutional responsibilities. Racism also manifested in a lack of service structure and support for Indigenous mental health worker roles. The racism was perceived to result in differential responses and treatment for Indigenous youth, which was described as inaction in the face of social need, an increased use of coercive practices, and a limited availability of Indigenous specific mental health workforce.

There is now recognition that it is not enough for mental health services to be non-racist but rather that an anti-racism approach is required (Cénat, 2020). Authors have theorized about the practical applications of anti-racist interventions in mental health care. The foundation of this is a critical awareness that racism is pervasive across policy and people (Cénat, 2020; Mensah et al., 2021) and that there needs to be a willingness to name and identify it in order to take anti-racist action (Cénat, 2020). These findings have highlighted target areas of organizational culture and structure for anti-racism efforts.

The inter-related nature of racism was evidenced particularly in regard to risk management culture, which was consistent with Nazroo et al. (2020) conceptualization of how interpersonal racism interacts with the other levels of racism through routine practices and collective organizational cultures. Despite evidence of high rates of false-positive categorization of risk, including violence (Large et al., 2011), risk assessment and management culture is a leading discourse that shapes the way services and treatments are delivered and how resources are prioritized (Crowe & Carlyle, 2003). The adverse consequences of this dominant culture, including coercive and disempowering practices, may be exacerbated for Indigenous youth by racism in the form of a perceived increased risk status. This perception is supported by the differences in use of compulsory treatment in New Zealand, with non-Indigenous people up to four times less likely to be placed under compulsory treatment orders than Indigenous people (Ministry of Health, 2021). Risk averse cultures within mental health settings are shaped through societal expectations for mental health services to manage risk associated with severe mental illness, which is reinforced by inquiry processes, and media portrayals of inadequate service responses that have resulted in adverse outcomes (Manuel et al., 2018). This creates a force that is difficult to overcome because ostensibly risk management creates a sense of safety for mental health institutions, people working within them, and society more generally. However, risk discourse may be inherently racist in that that those with more adverse social backgrounds are likely to be deemed higher risk, which is further reinforced with behavioural or aggressive racial stereotyping. This was particularly the case for male Indigenous youth who were perceived as vulnerable targets of risk related racism. Replacing the emphasis on risk management with a humanistic approach to treatment that tends to the real needs of people has been suggested as an appropriate anti-racist alternative approach to treatment (Cénat, 2020).

Participant experiences also revealed how institutional structures and cultures undermined the importance placed on social responsibility and Indigenous workforces. Generally, there is a high level of support for Indigenous mental health roles (O’Keefe et al., 2021), but research suggests a number of institutional factors counteract potential benefits. A systematic review of factors affecting the retention of Indigenous Australians in the health workforce, found poorly understood roles, high workloads and culturally unsafe workplaces were important factors (Lai et al., 2018). This is consistent with participant descriptions here. Furthermore, current mental health funding structures reflect colonial agendas and adequate support of these roles would require structural reorganization of resource within mental health settings in order to prioritize funding so that Indigenous roles could be delivered as intended by Indigenous communities. Similarly, an essential component of anti-racism efforts would include broadening cross-sector institutional scope to include a higher level of social responsiveness would also require re-prioritizing institutional expenditure and accountabilities.

## Strengths and limitations

The study has a number of strengths including its CRT design and commitment to adhering to Māori protocols and processes. This is also the first known study that privileges the voices of Indigenous people to explore how racism impacts early psychosis. The inclusion of Māori youth with FEP, families and Indigenous health professionals’ participants also provided a broad range of perspectives. Detailed information regarding the positioning of the researchers and methods have been provided to enhance interpretation and transferability of the results. Given this was a deductive structural analysis of a broader qualitative inquiry, participants were not asked specifically about experiences of racism and therefore the findings may not provide the full breadth of detail on the topic. There may be some limitations of transferability of the findings to other populations given the results were specific to Indigenous Māori. However, it is likely that aspects of the participant experiences are relevant to other populations given the heavy burden of racism known internationally.

## Conclusion

In order for the impact of racism on early psychosis to be dismantled, barriers to equity associated with racism need to be considered. This includes further investigation about how organizational cultures may differentially impact Indigenous and minority people, particularly in regard to social responsiveness, risk discourse and the distribution of workforce expenditure. These areas of organizational culture and structure are useful targets for anti-racism efforts. If the same level of onus and accountability was placed on institutions to achieve equity as there is on other organizational agendas such as risk mitigation, further progress may have been made to date.
